# Supplementary material for: Epidermal Fatty Acid-Binding Protein 5 (FABP5) Involvement in Alpha-Synuclein-Induced Mitochondrial Injury under Oxidative Stress
Source: Biomedicines. 2021 Jan 22;9(2):110. doi: 10.3390/biomedicines9020110 (PMC7911662; doi:10.3390/biomedicines9020110)

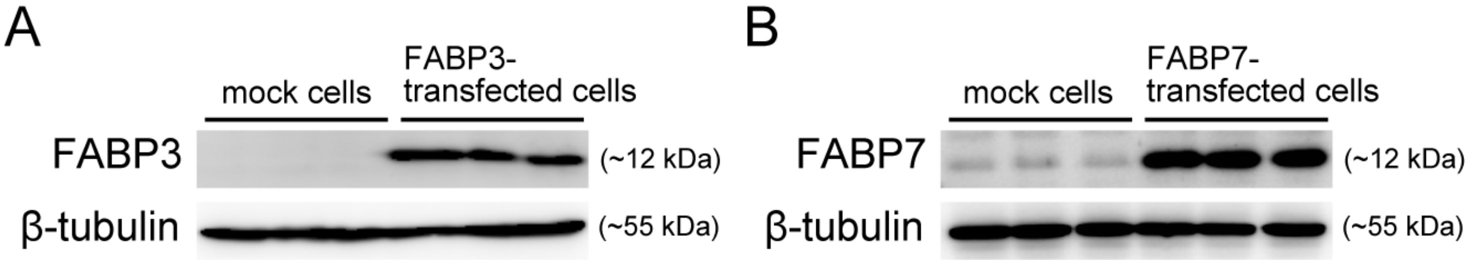

Figure S1. There is no FABP3 and a very little FABP7 expression in Neuro-2A cells (mock cells). (A-B) Representative images of immunoblots probed with antibodies against FABP3 (1:200; 10676-1-AP, ProteinTech, Chicago) and FABP7 (1:200; AF3166, R&D Systems, Minneapolis, MN, USA). Blots with anti-β-tubulin antibody showed that a similar amount of protein was loaded.

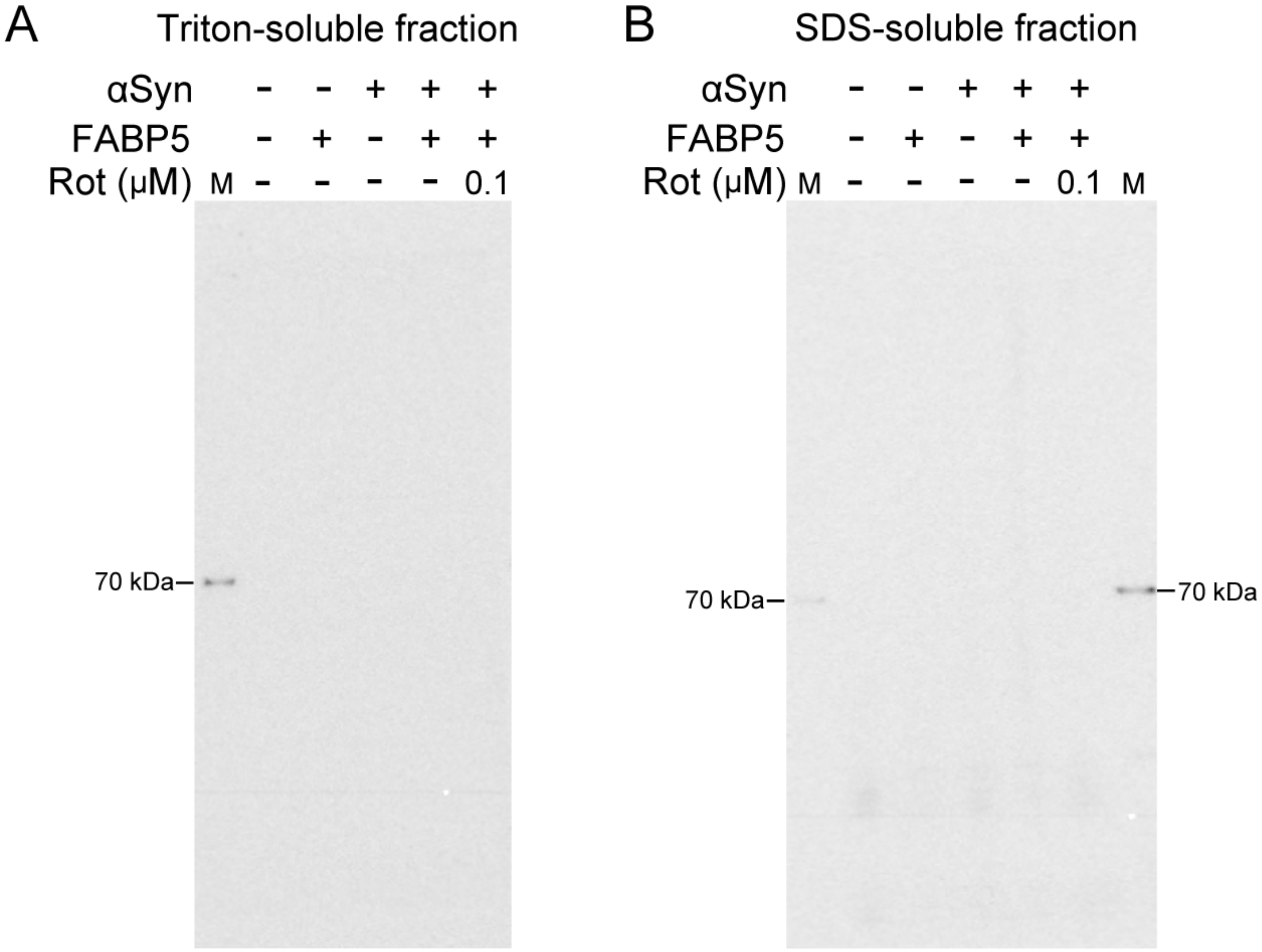

Figure S2. The negative control of immunoblotting assay. PVDF membranes were incubated without primary antibody and subsequently probing with anti-mouse IgG secondary antibody. And there is no target band detected. M: marker protein (SS-2000, Cosmo Bio Co., Ltd., Tokyo, Japan).

Figure 1 - original images

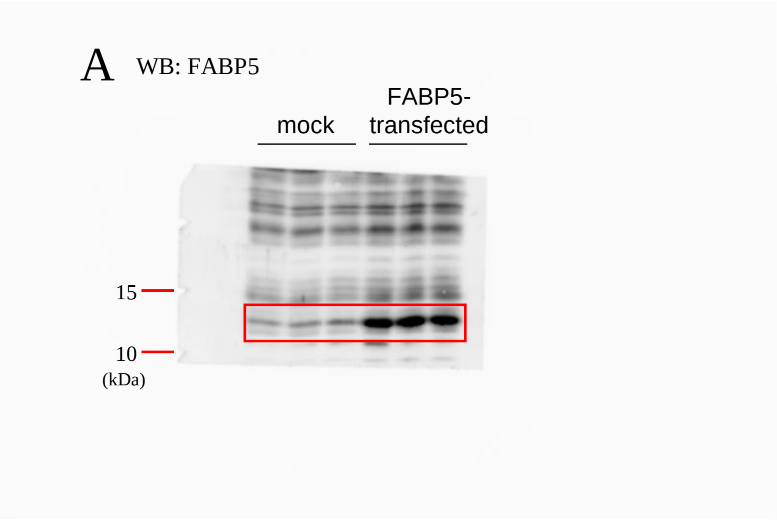

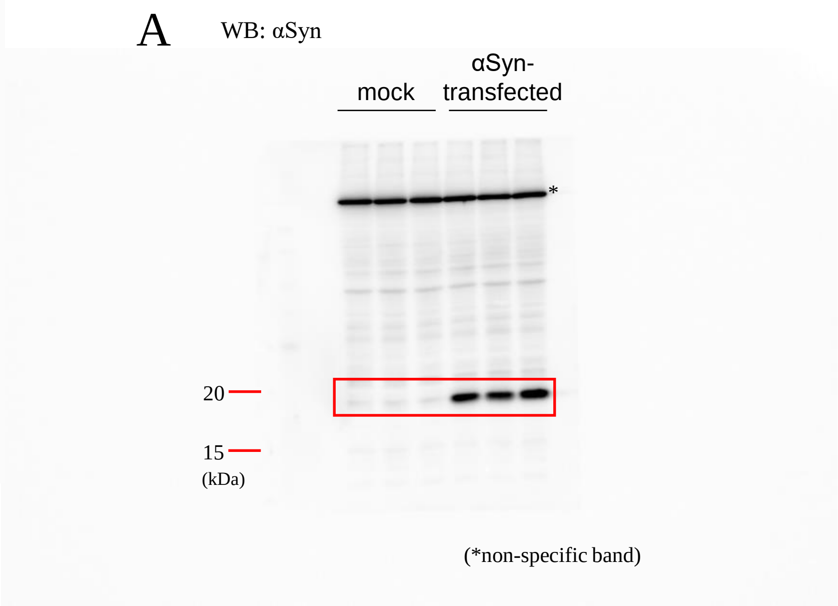

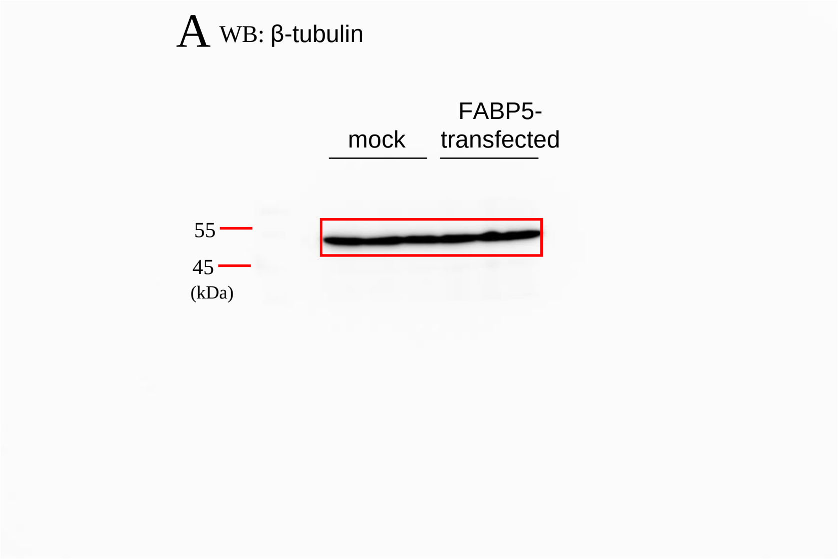

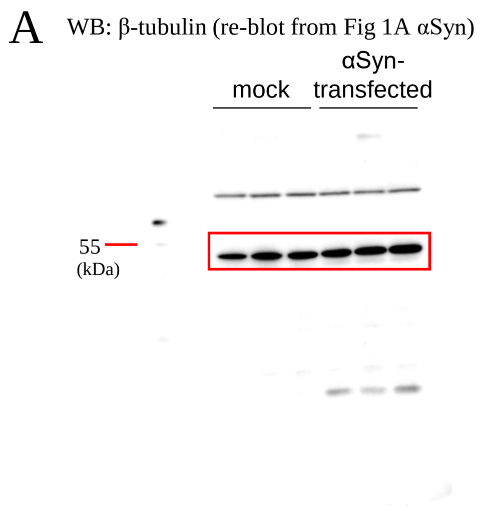

Figure 2 - original images

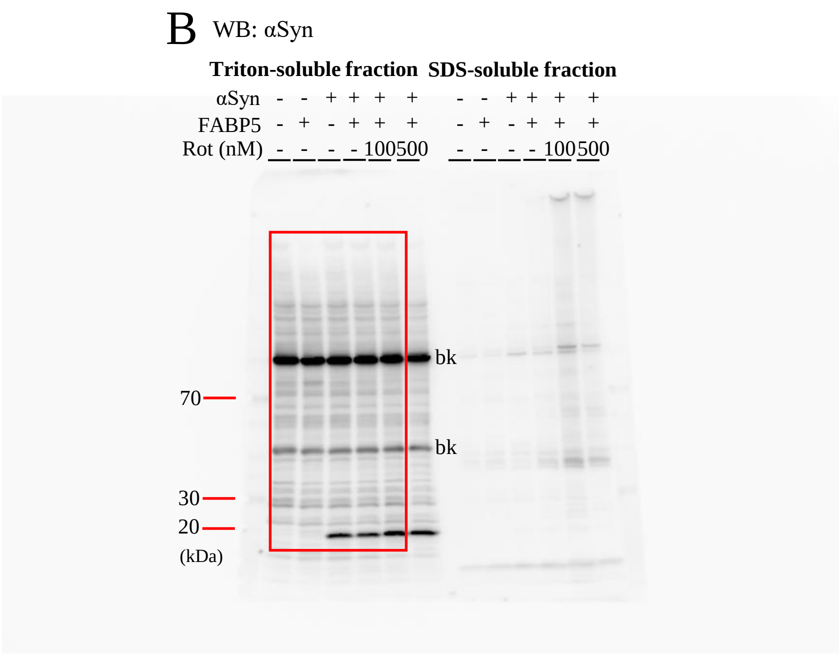

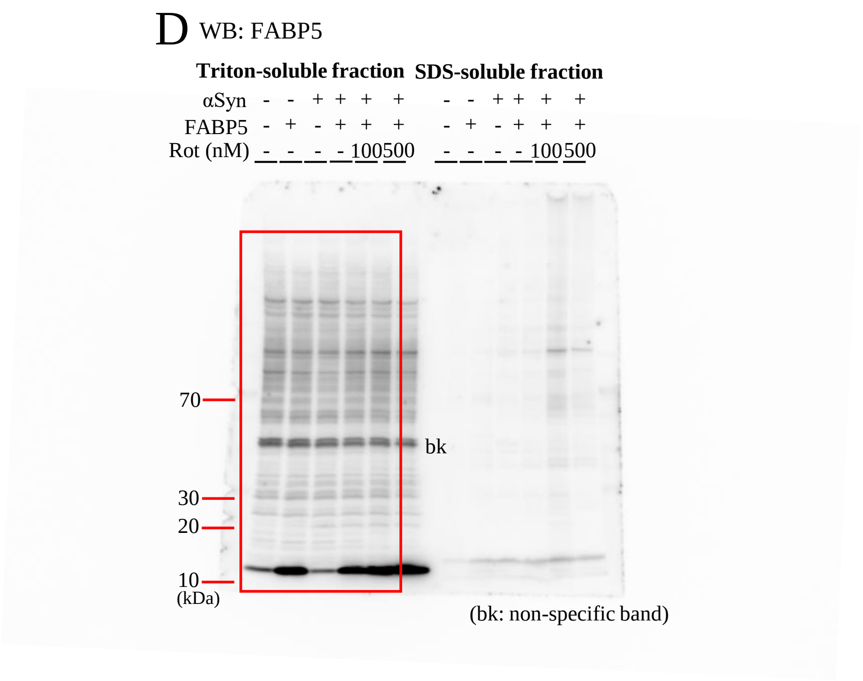

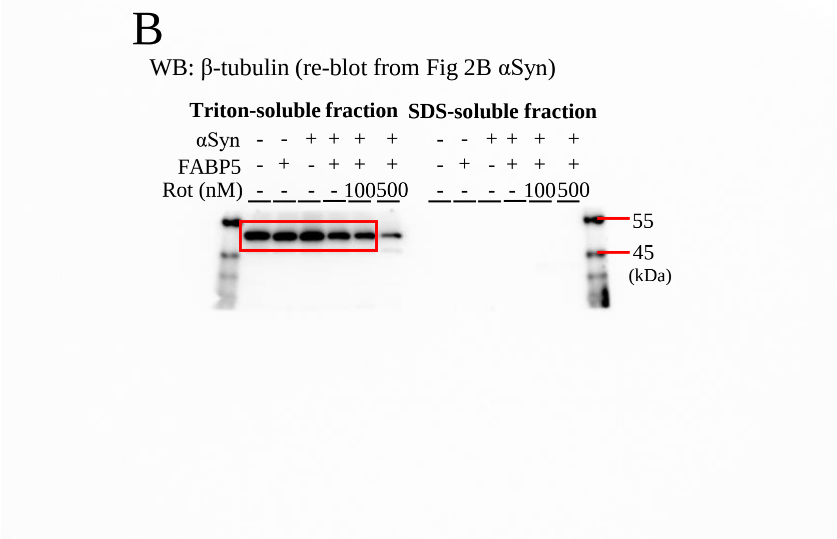

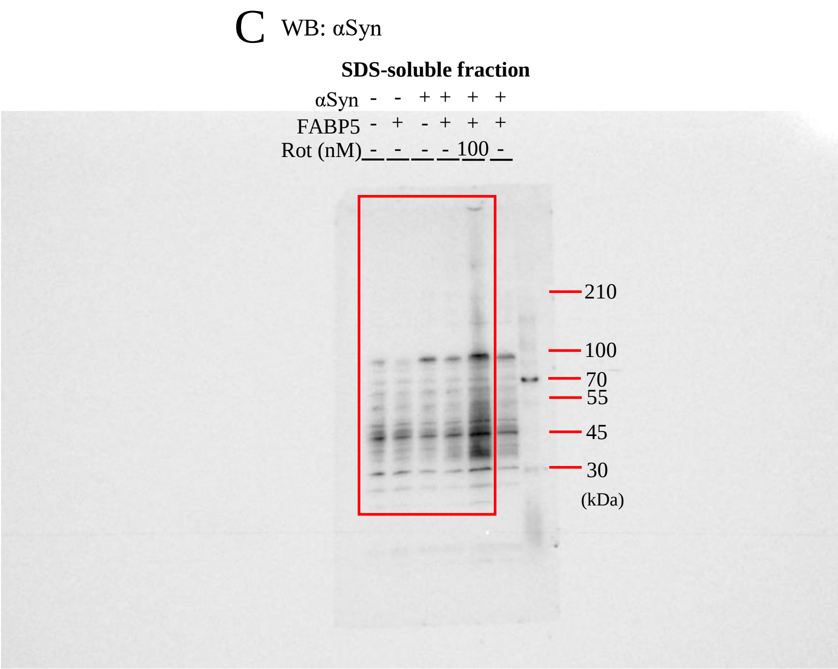

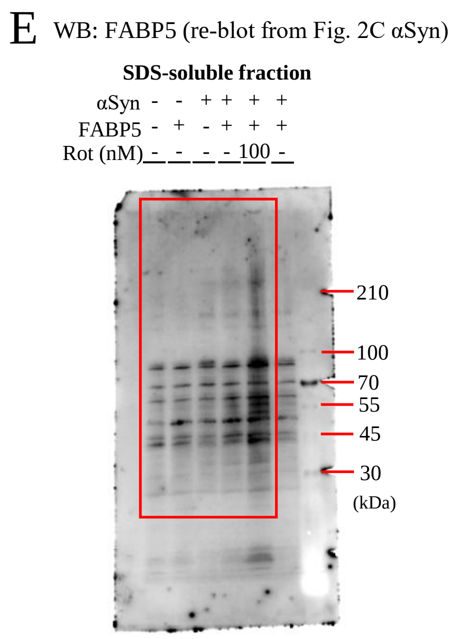

Figure 5 - original images

E WB: αSyn

SDS-soluble fraction

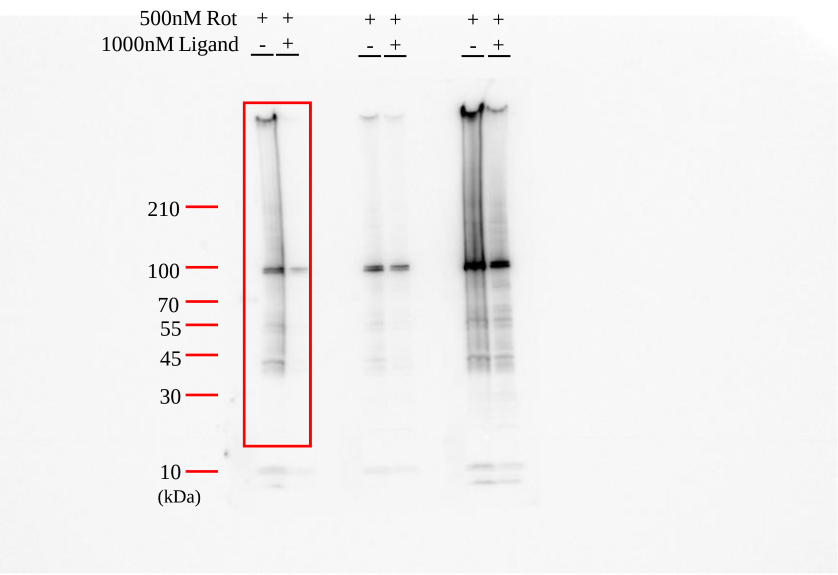

F WB: FABP5 (re-blot from Fig. 5E αSyn)

SDS-soluble fraction

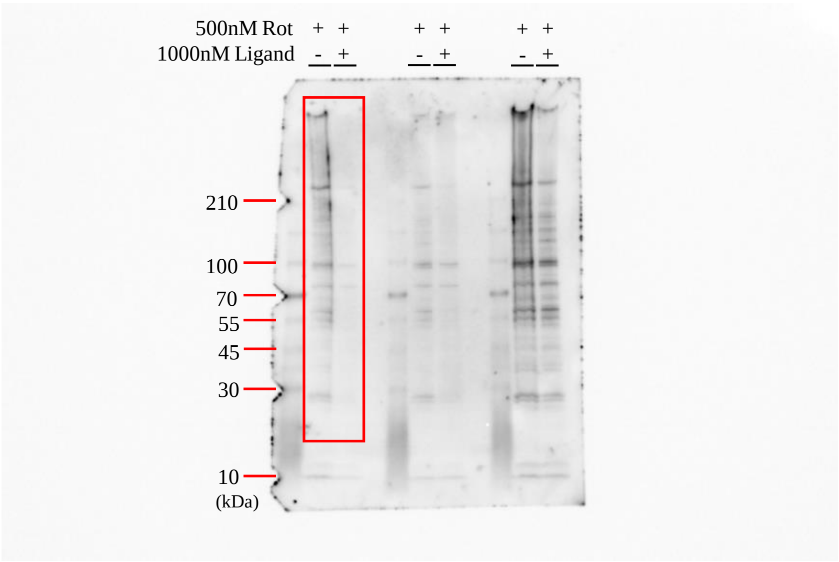

G WB: β-tubulin

Triton-soluble fraction

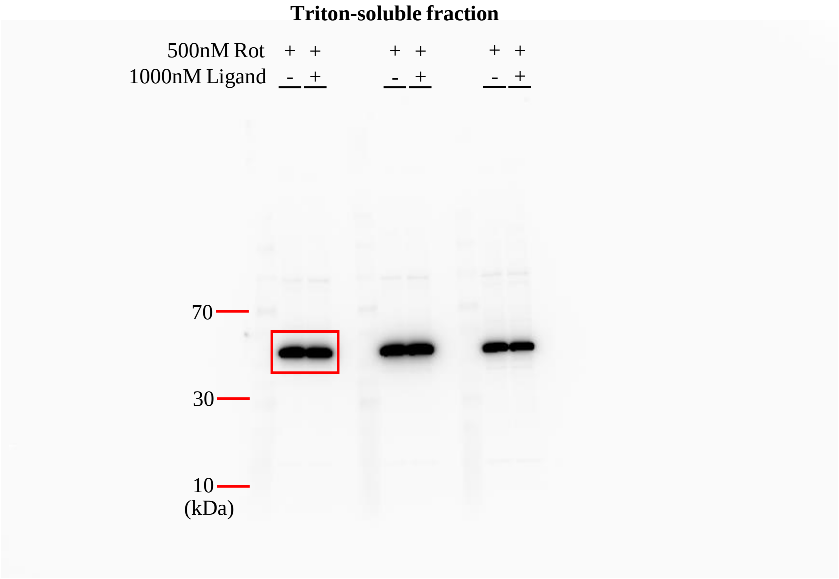

G WB: αSyn (re-blot from Fig. G β-tubulin)

Triton-soluble fraction

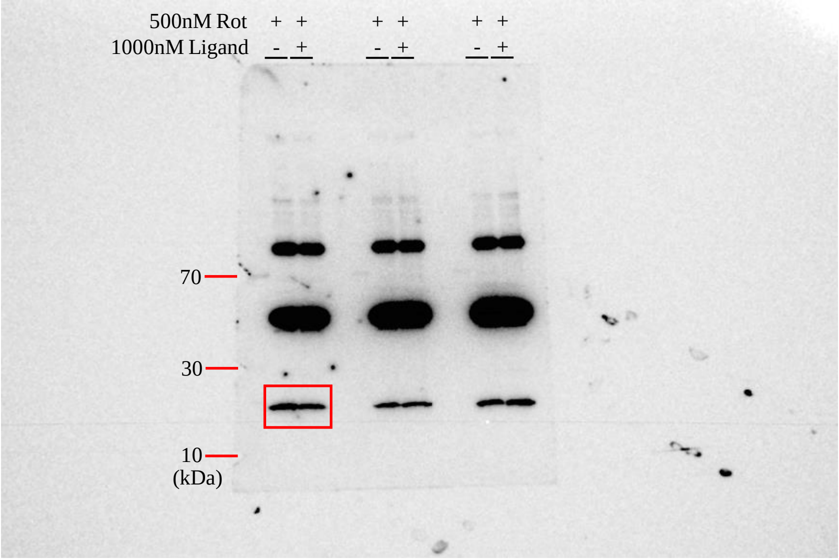

G WB: FABP5 (re-blot from Fig. G αSyn)

Triton-soluble fraction

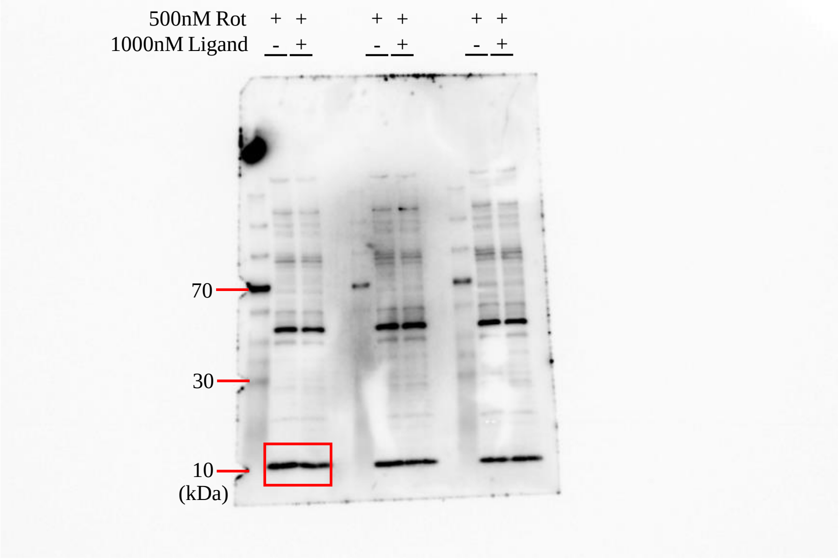

Figure 6 - original images

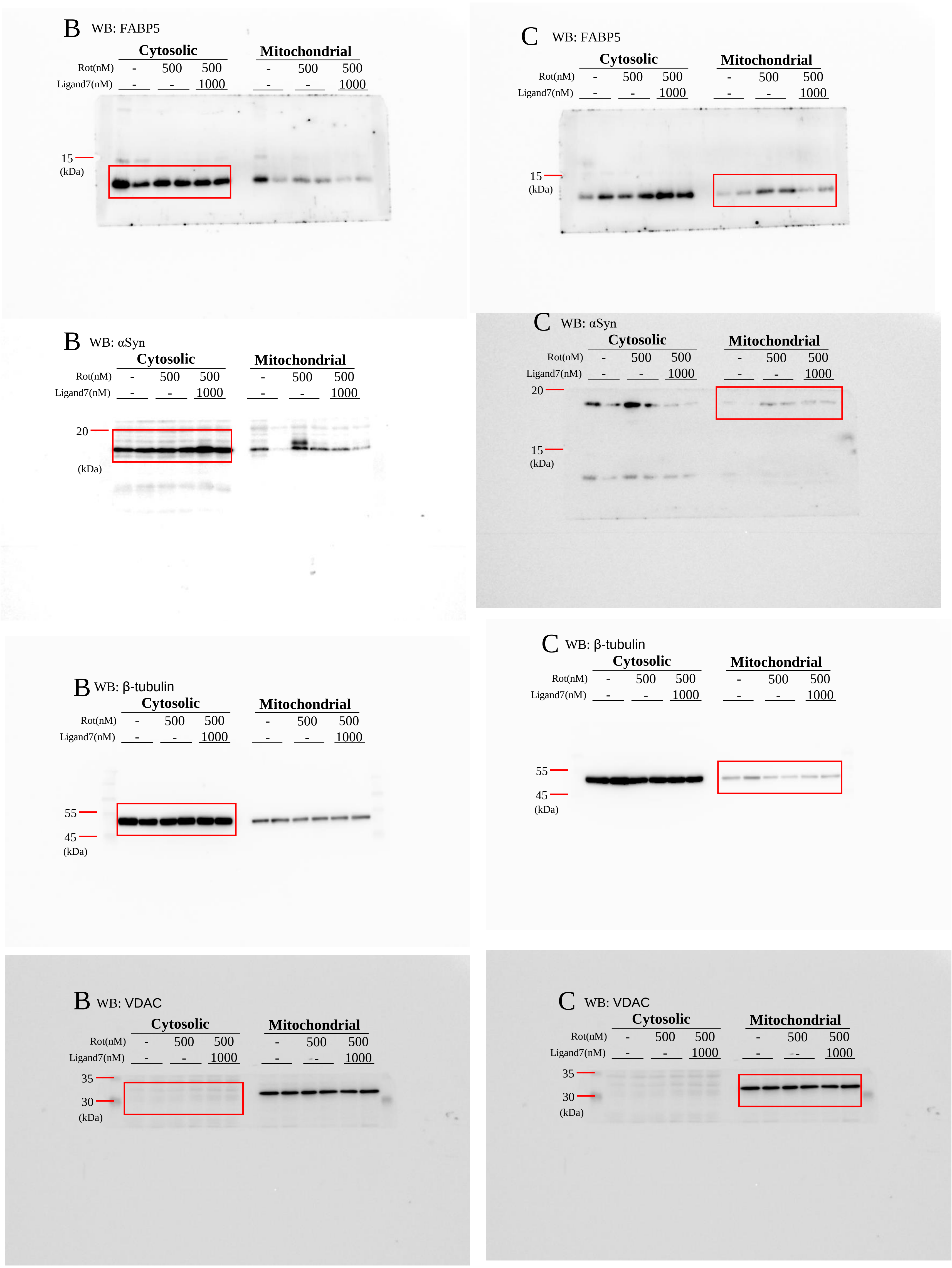

Figure S1 - original images

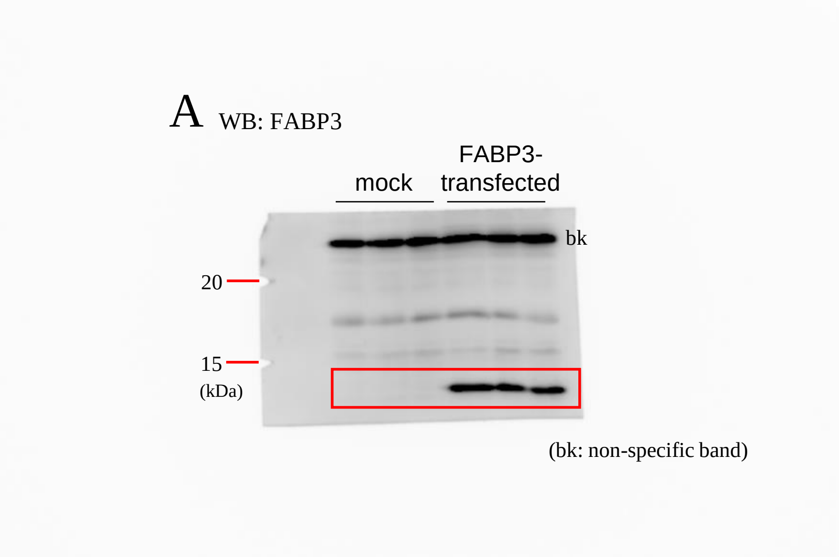

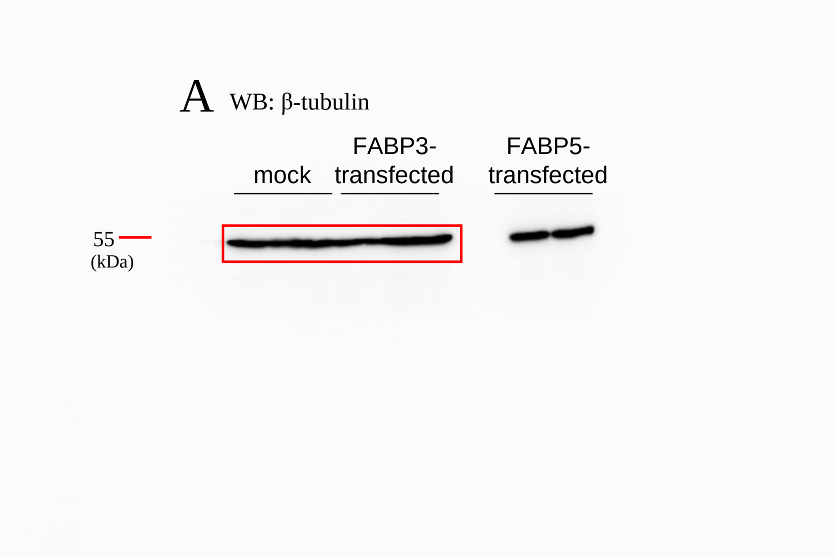

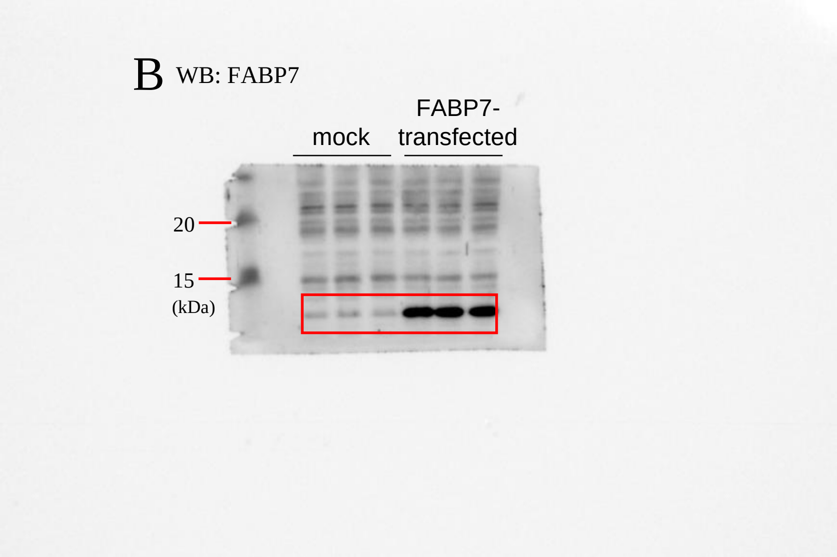

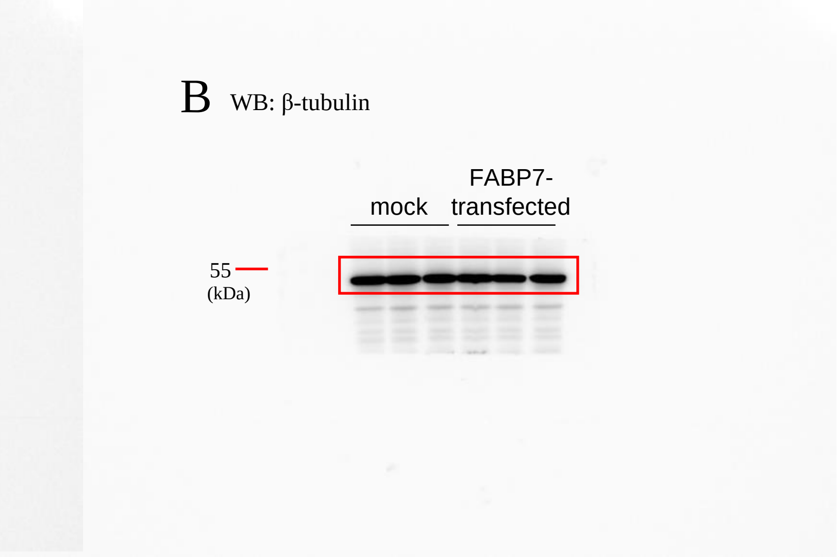

Figure S2 - original images

WB: without primary antibody and subsequently probing with anti-mouse IgG secondary antibody. (pl: protein ladder)

A **Triton-soluble fraction**

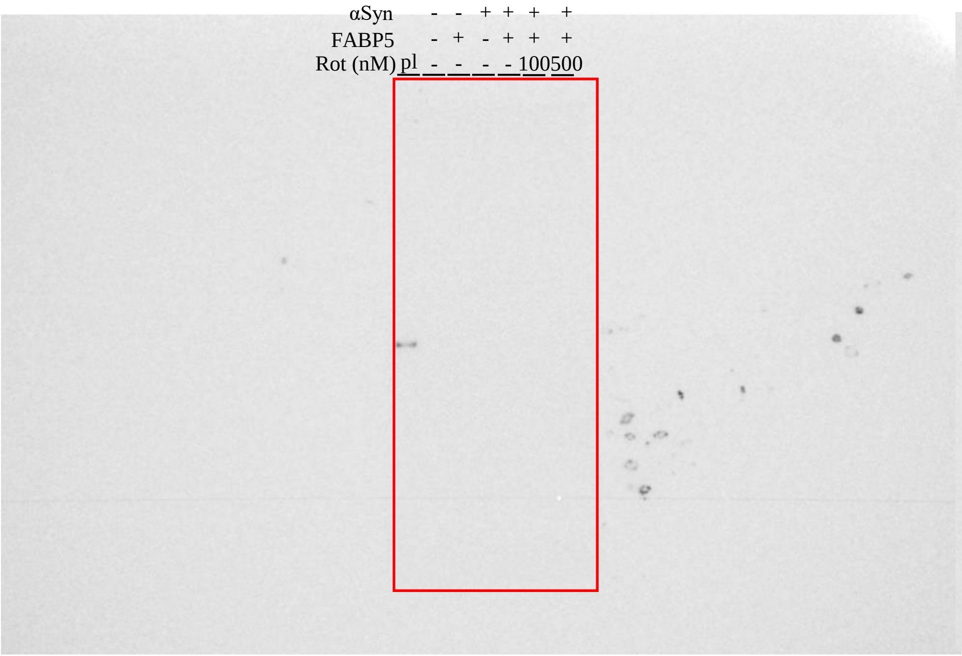

B **SDS-soluble fraction**

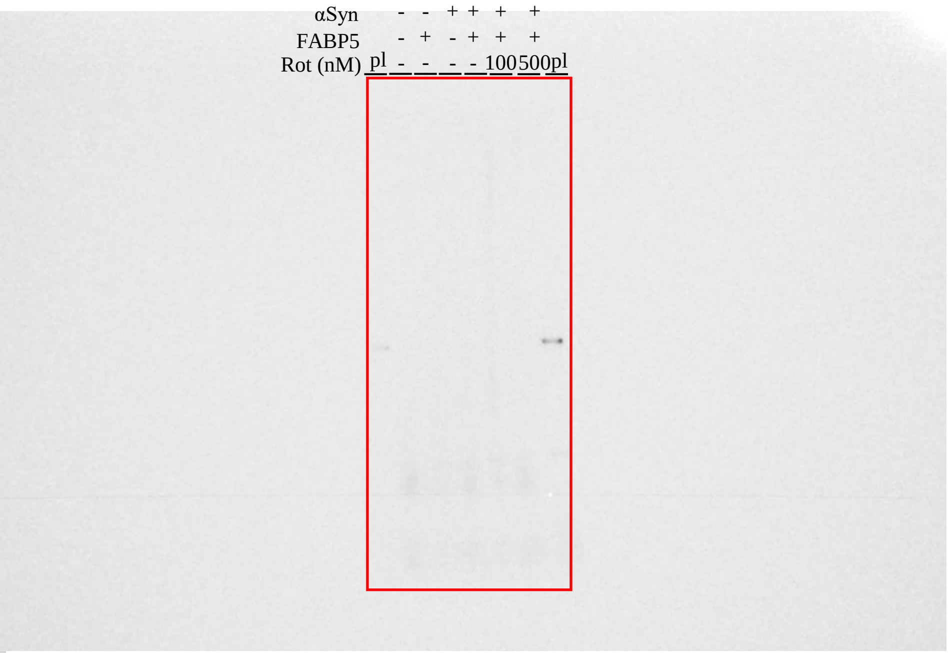

Supplement: Supplementary file 1 [file biomedicines-09-00110-s001.pdf]
